# The Importance of Subjective Cognitive Decline Recognition and the Potential of Molecular and Neurophysiological Biomarkers—A Systematic Review

**DOI:** 10.3390/ijms241210158

**Published:** 2023-06-15

**Authors:** Janina Ulbl, Martin Rakusa

**Affiliations:** 1 Division of Neurology, University Medical Centre Maribor, 2000 Maribor, Slovenia; janina.ulbl@gmail.com; 2 Faculty of Medicine, University of Maribor, 2000 Maribor, Slovenia

**Keywords:** Alzheimer’s disease, electroencephalography, event-related potentials, mild cognitive impairment, subjective cognitive decline

## Abstract

Subjective cognitive decline (SCD) and mild cognitive impairment (MCI) are early stages of Alzheimer’s disease (AD). Neurophysiological markers such as electroencephalography (EEG) and event-related potential (ERP) are emerging as alternatives to traditional molecular and imaging markers. This paper aimed to review the literature on EEG and ERP markers in individuals with SCD. We analysed 30 studies that met our criteria, with 17 focusing on resting-state or cognitive task EEG, 11 on ERPs, and two on both EEG and ERP parameters. Typical spectral changes were indicative of EEG rhythm slowing and were associated with faster clinical progression, lower education levels, and abnormal cerebrospinal fluid biomarkers profiles. Some studies found no difference in ERP components between SCD subjects, controls, or MCI, while others reported lower amplitudes in the SCD group compared to controls. Further research is needed to explore the prognostic value of EEG and ERP in relation to molecular markers in individuals with SCD.

## 1. Introduction

Alzheimer’s disease is a progressive neurodegenerative disease that significantly impacts patients’ lives. It is the most common type of dementia, accounting for more than 60 % of cases [1]. Pathophysiological changes in AD occur decades before the onset of clinical symptoms and signs [2]. Pre-dementia stages are subjective cognitive decline (SCD) and mild cognitive impairment (MCI). SCD is described as a self-experienced decline in cognitive capacity compared to an individual’s previous level of functioning, while the performance on standardised cognitive tests is normal [3].

The diagnosis of AD is based on pathological amyloid and tau biomarkers independent of clinical symptoms [4]. Biomarker abnormalities can also be detected in the SCD and MCI stages and are related to clinical progression. In five years, 7.2% of SCD patients progress to dementia and 20.8% of SCD patients develop MCI. The relative risk of dementia for patients with SCD is 2.17 relative to those without SCD [5].

Developing novel biological treatments for AD requires the recognition of the cognitive decline in its early stages to effectively intervene and slow down the progression of the disease [6]. Therefore, identifying reliable and accessible biomarkers is essential for the early detection and monitoring of AD. Currently, cerebrospinal fluid (CSF) biomarkers are limited due to their invasive nature, while serum biomarkers and amyloid PET-CT are associated with high costs [4,7]. Therefore, neurophysiological biomarkers present a promising alternative, as they are non-invasive, relatively inexpensive, and do not involve radiation exposure [8].

One such neurophysiological biomarker is electroencephalography (EEG), which measures electrical currents or potentials generated by cortical neurons [9]. Alterations in neural electrical potentials can be directly detected using EEG. Spectral analysis of EEG signals in AD patients has revealed a decrease in power in the alpha (8–15 Hz) and beta (16–31 Hz) bands, coupled with an increase in power in the theta (4–8 Hz) and delta (0.5–4 Hz) bands [10]. Although EEG offers high temporal resolution, its spatial resolution is relatively low. To overcome the spatial resolution limitations of EEG, standardised low-resolution brain electromagnetic tomography (sLORETA) can be employed. Analysing multiple EEG signals, sLORETA allows us to determine their cortical source, thus demonstrating the spatial dynamics of brain activity [11]. In AD patients, changes in the power of EEG bands are typically observed in temporal and central frontal areas, and these changes correlate with neuropsychological test results and imaging biomarkers [10].

Another neurophysiological biomarker is event-related potentials (ERPs). ERPs are small voltages generated by cortical neurons in response to sensory, motor, or cognitive stimuli [12], and are a widely used tool to assess cognitive processing with high temporal resolution. Cognitive ERPs have been widely utilised to study dementia and ageing, as the P300 ERP component is easily observed and reflects attention and memory processing [13]. Observed changes in AD patients are decreased amplitude and increased latency of typical ERP components [14].

EEG patterns and ERP changes observed in AD patients have also been reported in individuals with MCI [10,15]. Moreover, disruptions in functional connectivity, characterised by the loss of connection between different brain regions, could contribute to the cognitive decline associated with AD [16].

While ERPs’ sensitivity in differentiating individuals with SCD and MCI remains less known, early evidence indicates similar brain alterations between these two groups [17]. SCD can be related to disorders other than AD, such as psychiatric diseases, and individual diagnostic workups may help reveal underlying causes [18].

Therefore, the study of ERPs in the context of SCD could provide valuable insights into the early detection of cognitive decline and the development of interventions to slow or prevent the progression of cognitive impairment.

Our study aimed to thoroughly analyse EEG and ERP use in diagnosing SCD in AD patients, seeking a clearer understanding of their effectiveness in identifying cognitive decline and assessing their potential to improve early diagnosis and treatment strategies.

## 2. Materials and Methods

The systematic literature search was conducted in March 2023 according to the Preferred Reporting Items for Systematic Reviews and Meta-Analyses (PRISMA) guidelines [19]. PubMed and Web of Science databases were used. We ran nine sets of searches with different keywords (Table 1) separated by “AND” and “OR” Boolean operators. We first removed the duplicates. Afterwards, we excluded the successive types of studies: reviews, case reports, conference abstracts, and study designs.

The inclusion criteria were the following: (I) participants had SCD with normal performance on standard neuropsychological tests; (II) a specific EEG or ERP parameter was measured; (III) the article was published between January 2011 and March 2023; (IV) full text was available in English. In addition, we excluded studies not related to our topic based on their title or abstract. Those were mostly studies that investigated not EEG/ERP but other types of biomarkers or did not include SCD participants but MCI or AD patients. A few studies were discarded after full-text analysis for not meeting our inclusion criteria (for example, magnetoencephalography was used instead of EEG).

**Figure 1:**
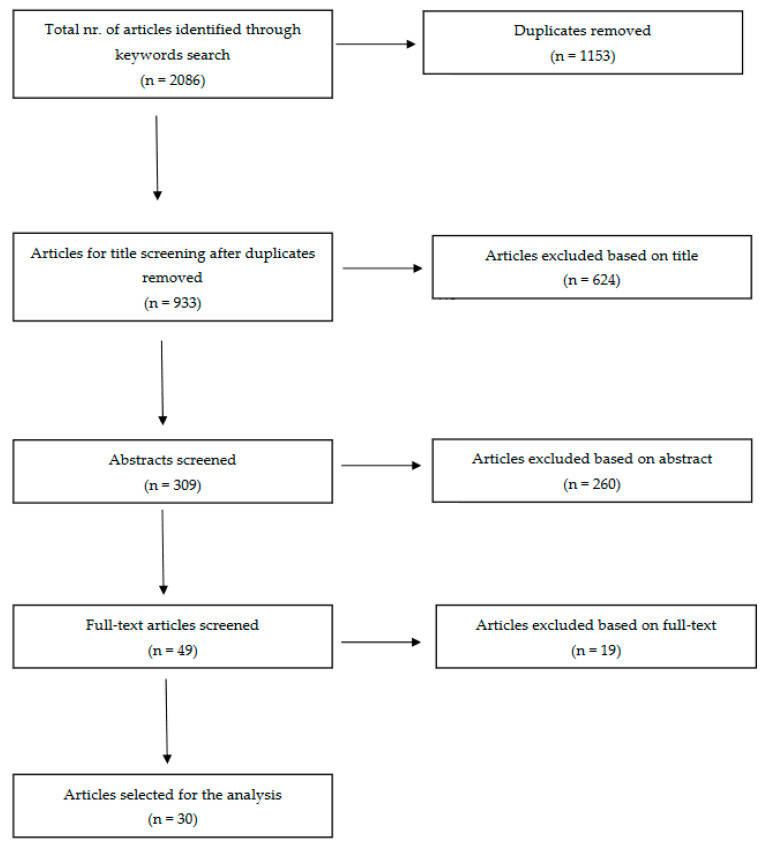
Preferred Reporting Items for Systematic Reviews and Meta-Analyses (PRISMA) literature review flow diagram.

The detailed search protocol is shown in Figure 1.

## 3. Results and Discussion

The search results yielded 2086 studies across various keyword combinations in two databases (Table 1). After eliminating duplicates, 933 articles underwent title screening, from which 309 advanced to abstract evaluation and 49 to full-text assessment (Figure 1). Ultimately, 30 articles were selected for inclusion in this review. A summary of the main findings and limitations for each article can be found in Table 2 and Table 3.

For this discussion, we reviewed the existing literature and selected 30 papers on EEG/ERPs changes in patients with SCD and compared these findings to those observed in patients with MCI, AD, and cognitively unimpaired controls. We furthermore compared EEG/ERP biomarkers to the established fluid, neuroimaging, and genetic biomarkers.

**Table 2:** EEG findings in patients with subjective cognitive impairment, mild cognitive impairment, and Alzheimer’s disease.

Reference	Participants	SCD Measure	Task	Observed Parameters	Main Findings	Main Limitations
Sibilano et al., 2023 [20]	118: SCD (56), MCI (45), controls (17)	SCD-I criteria [3]	Resting-state	Theta, alpha, beta, and delta frequency bands	Delta frequency bands are the most discriminative for SCD and MCI	Small sample; Usage of the novel deep learning approach
2. Zheng et al., 2023 [21]	53: SCD (25), controls (28)	Nine-item Subjective Cognitive Decline Questionnaire [22]	Resting-state	Theta frequency band	Relative theta power: SCD > controls in left frontal region	Small sample
3. Abazid et al., 2022 [23]	102: SCD (22), MCI (52), AD (28)	SCD-I criteria [3]	Resting-state	Functional connectivity (EpEn; MSC; PLI; Clustering coefficient; Shortest path)	EpEn and clustering coefficient differences; Stronger long-term connectivity in SCD	Resting-state only; Small sample
4. Ganapathi et al., 2022 [24]	161: SCD (69), MCI (53), AD (39)	Cognitive complaints	Resting-state	Peak-alpha frequency; Theta/beta ratio	No significant group differences	Lack of control group
5. Lazarou et al., 2022 [25]	73: SCD (17), MCI (23), AD (12), controls (21)	SCD-I criteria [3]	Visual attention and short-term memory task (baseline and 3 years follow-up)	Functional connectivity (Clustering coefficient; Strength; Betweenness centrality)	Controls: denser network compared to other groups; SCD converted to MCI vs. stable SCD: lower values of all functional connectivity measures at the baseline	Small sample; Lack of functional connectivity measures at the follow-up
6. Shim et al., 2022 [26]	95: SCD Aβ+ (26), SCD Aβ− (69)	SCD-I criteria [3]	Resting-state	Delta, theta, alpha, beta, and gamma frequency bands	SCD Aβ+ vs. SCD Aβ−: increased relative delta power and decreased alpha activity	No control group; Lack of longitudinal data
7. Iliadou et al., 2021 [27]	76: SCD (43), MCI (33)	Not reported	Resting-state; Game-based test	Alpha, beta, delta, and theta frequency bands	SCD < MCI in alpha, beta, and theta bands (resting and task)	Small sample; More females
8. Babiloni et al., 2020 [28]	172: SCD Aβ− (118) SCD, Aβ+ (54)	Memory complaints	Resting-state	Alpha frequency band	SCD+ vs. SCD−: occipital alpha2 and temporal alpha3 rhythm differences	Short EEG (1 min); only 19 electrodes
9. Briels et al., 2020 [29]	809: SCD (399), AD (410)	Not reported	Resting-state	Functional connectivity (PLI)	SCD vs. AD: connectivity differences	No external validation group
10. Engedal et al., 2020 [30]	213: SCD (47), MCI (99), controls (67)	SCD-I criteria [3]	Resting-state	Alpha, beta, theta, and delta frequency bands; SPR; DI	Baseline DI differences; 35% conversion; DI: converters > non-converters	Misdiagnosis possibility
11. Gaubert et al., 2019 [31]	318 with SCD: Aβ+ N+ (25), Aβ+ N− (63), Aβ− N+ (51), Aβ− N− (175)	Not reported	Resting-state	Functional connectivity (PSD; MSF; SE; wSMI)	Non-linear relationship between FC measures and amyloid load	No tau protein measured
12. Dubois et al., 2018 [32]	318: SCD Aβ+ (88), SCD Aβ− (230)	15-item McNair questionnaire	Resting-state; Cognitive task	Alpha:theta ratio and alpha frequency band	Decreased alpha:theta ratio; Increased prefrontal alpha (24 months) in Aβ+ group	No control group; Short follow-up
13. Houmani et al., 2018 [33]	169: SCD (22), MCI (58), AD (49), other pathologies (40)	SCD-I criteria [3]	Resting-state	EpEn; EEG local synchrony	EpEn: SCD > AD; Theta synchrony: SCD < AD	Small sample size
14. Mazzon et al., 2018 [34]	26: SCD (8), MCI (11), controls (7)	Subjective memory complaints	Resting-state; Mental memory task	IAF peak; alpha 1, beta 1, and gamma frequency bands; FD	SCD: lower frontal IAF peak, parietal FD, beta 1 and 2, gamma; Alpha power reduction during task: frontal in controls and SCD, diffused in MCI	Small sample size
15. Smailovic et al., 2018 [35]	637: SCD (210), MCI (230), AD (197)	SCD-I criteria [3]	Resting-state	Functional connectivity (GFP; GFS); CSF biomarkers	Differences in GFP and GFS; CSF-GFP/GFS correlations	No comparison with controls
16. Teipel et al., 2018 [36]	318 SCD: Aβ+ (63), Aβ− (252)	Memory complaints (6 + months)	Resting-state	Cortical amyloid load (florbetapir-PET); Functional connectivity (PLI)	No amyloid load-PLI associations	No controls or AD comparison
17. Gouw et al., 2017 [37]	205 amyloid positive: SCD (63), MCI (142)	SCD-I criteria [3]	Resting-state	Alpha, beta, delta, and theta frequency bands	No global EEG predictors; SCD: higher delta and theta, lower alpha power related to clinical progression	3 different EEG systems
18. Yu et al., 2016 [38]	181: SCD (64), AD (69), FTD (48)	Not reported	Resting-state	Functional connectivity (PLI; MST)	PLI and global efficiency differences: SCD, FTD, AD	No comparison with non-SCD controls
19. Kramberger et al., 2013 [39]	755: SCD (310), MCI (285), AD (131), mixed dementia (29)	Subjective complaints	Resting-state	BA; EA; CSF markers	BA, EA frequency differences; CSF-BA/EA associations	Small sample; Mixed dementia overlap

Abbreviations: Aβ: Amyloid Beta; AD: Alzheimer’s Disease; BA: Background Activity; CSF: Cerebrospinal Fluid; DI: Dementia Index; EA: Episodic Abnormalities; EEG: Electroencephalography; EpEn: Epoch-based Entropy; FC: Functional Connectivity; FD: Fractal Dimension; FTD: Fronto-temporal Dementia; GFP: Global Field Power; GFS: Global Field Synchronization; IAF: Individual Alpha Frequency; MCI: Mild Cognitive Impairment; MSC: Magnitude Squared Coherence; MSF: Median Spectral Frequency; MST: minimum Spanning Tree; N: Neurodegeneration positive; PET: Positron Emission Tomography; PLI: Phase Lag Index; PSD: Power Spectral Density; qEEG: Quantitative EEG; SCD: Subjective Cognitive Decline; SE: Spectral Entropy; SPR: Statistical Pattern Recognition; t-tau: Total Tau; p-tau: Phosphorylated Tau; wSMI: Weighted Symbolic Mutual Information.

### 3.1. Resting-State EEG Spectral Analysis

The majority of analysed papers have reported abnormal resting-state EEG activity in participants with SCD compared to MCI, AD, and normal controls (Table 2). Resting-state EEG refers to the measurement of brain activity during a relaxed state when the individual is not actively engaged in a specific cognitive task [40].

In a study by Babiloni et al. [28], resting-state posterior alpha rhythms were found to be abnormal in SCD seniors with preclinical Alzheimer’s neuropathology and high education levels. The study reported that higher-educated SCD participants with amyloid pathology had lower occipital alpha 2 power density compared to lower-educated SCD participants with amyloid pathology. Similarly, Gouw et al. [37] found that higher delta and theta power and lower alpha power were related to clinical progression in the SCD group. Accordingly, the most prevalent EEG spectral findings in AD patients involve decreased power in the alpha and beta bands and increased power in the theta and delta frequency bands [10].

In a study by Iliadou et al. [27], mean power in alpha, beta, and theta bands during resting state was lower in SCD participants than in those with MCI. Additionally, Smailovic et al. [35] reported that global field power (GFP) in delta and theta frequency bands was lower in SCD participants compared to AD, while GFP in alpha frequency bands was higher. Another study [21] found higher theta power in SCD compared to controls in the frontal region. Furthermore, the delta frequency band was reported [20] as the most discriminative for distinguishing SCD and MCI from controls.

These findings suggest that resting-state EEG changes in alpha, delta, and theta frequency bands may be indicative of early neuropathological changes in SCD individuals. In the examined studies, Babiloni et al. [28] noted that elevated posterior alpha 2 and increased temporal alpha 3 might act as neuroprotective elements in highly educated SCD participants compared to their less educated counterparts. Dubois et al. [32] and Gaubert et al. [31] also contended that the rise in high-frequency oscillations and the decline in low-frequency oscillations could result from compensatory activation in the frontal lobe. This concept is consistent with Alzheimer’s disease modelling research [41], which implies that compensatory mechanisms are responsible for preserving the baseline activity of alpha and theta oscillations. Furthermore, in MCI and AD patients, the majority of compensatory structural network changes were found predominantly in the frontal and temporal lobes [42], and could be valuable in characterising the progression of cognitive decline.

### 3.2. Functional Connectivity Measures

The normal human brain functional connectivity network is described as a “small-world” network. However, in AD, there is a shift towards “random-world” network properties, supporting the disconnection hypothesis [43]. Functional connectivity (FC) is an essential aspect of the analysis of resting-state EEG data, which has been investigated in eight out of 30 reviewed studies (Table 2).

FC measures represent statistical associations among spatially distant brain regions, reflecting neural communication and synchronisation patterns [44]. These measures included epoch-based entropy (EpEn), phase lag index (PLI), mean global coherence (Coh), phase locking value (PLV), amplitude envelope correlation (AEC), and others [44].

Briels et al. [29] suggested that AEC is the most robust and replicable estimate of functional connectivity in cognitive decline due to AD among the various FC measures. Several studies [23,29,33,38] have reported higher values of FC measures in alpha and beta frequency bands and lower values in theta and delta frequency bands when comparing subjects with SCD to AD patients (Table 2). Similar patterns have been observed when comparing AD and MCI subjects to cognitively unimpaired participants [45].

Two studies compared three stages of the AD disease spectrum: SCD, MCI, and AD [23,25]. They reported differences in FC parameters between diagnostic groups and observed stronger long-term connectivity between brain regions in SCD subjects than in MCI and AD patients. They also noted lower values of FC measures in those SCD participants who progressed to MCI. These findings suggest that subtle alterations in brain FC networks may already exist at the very early stages of cognitive decline and may also have a prognostic value.

### 3.3. Event-Related Potentials

In Table 3, we described 13 studies investigating ERPs in SCD participants. These studies employed various cognitive tasks and assessed a range of ERP components, including N170, P300, N200, late positive potential (LPP), median frontal negativity (MFN), feedback-related negativity (FRN), and mismatch negativity (MMN).

The N170 component, associated with face processing, has been investigated in two studies [46,47]. Lazarou et al. [46] found that the N170 amplitude was reduced in SCD patients compared to controls, with a pattern similar to MCI and AD patients. This suggests that face processing may be affected in the early stages of cognitive decline. However, Perez et al. [47] found only significant differences in N170 amplitude or latency between male patients with SCD and male controls. This discrepancy could be due to methodological differences, such as using different emotional valence stimuli.

The N200 and P300 components, linked to sensory processing, attention, working memory, and execution, were examined in nine studies (Table 3).

Some studies found no significant differences in P300 amplitude or latency between SCD patients and controls [17,47,48]. However, Smart et al. [49] found a reduced P300 amplitude in SCD patients, suggesting a decline in attention capacity. Zheng et al. [21] noted a small difference in the P300 effect when comparing the SCD group to the controls. A decrease in P300 amplitude was reported with more severe cognitive decline when comparing SCD, MCI, and AD diagnostic groups [24]. Another study [50] found that SCD participants with high memory complaints had reduced N200-P300 peak-to-peak amplitudes compared to those with low memory complaints. This implies that the severity of SCD may influence ERP changes.

The MMN was investigated in three studies with inconsistent results [51,52,53]. Hagen et al. [53] found that SCD patients exhibited increased P200 latency compared to controls, whereas Idrizbegovic, Hederstierna, and Rosenhall [52] reported a reduced MMN amplitude in SCD patients, similar to AD patients. Laptinskaya et al. [51] found that the duration of the MMN change was positively associated with episodic memory performance, suggesting a potential link between auditory memory decay and cognitive decline.

**Table 3:** Event-related potential findings in patients with subjective cognitive impairment, mild cognitive impairment, and Alzheimer’s disease.

Reference	Participants	Measure of SCD	Task	Observed Parameters	Main Findings	Main Limitations
Zheng et al., 2023 [21]	53: SCD (25), Controls (28)	Nine-item Subjective Cognitive Decline Questionnaire (SCD-Q9)	Delayed match-to-sample task	ERP P300 amplitude and effect	Match-related P300 effect: SCD > controls in right frontal region	Small sample
2. Ganapathi et al., 2022 [24]	161: SCD (69), MCI (53), AD (39)	Cognitive complaints	Go/NoGo task	ERP P300 amplitude and latency	P300 amplitude: SCD > MCI > AD	Lack of control group
3. Garrido-Chaves et al., 2021 [48]	136: young SCD (28), young no SCD (37), older SCD (31), older no SCD (39)	MFE-30 Questionnaire	Iowa Gambling Task	ERP P300 and FRN amplitude and latency	Older SCD worse in ambiguity phase, FRN latency differences	Highly educated participants
4. Perez et al., 2021 [47]	79: SCD (41), Controls(38)	MFE-30 Questionnaire	Facial affect labelling	ERP P300, N170, LPP amplitude and latency	Mostly similar between SCD and controls; longer N170 latency in men with SCD	Highly educated participants; Stationary stimuli
5. Susana, Mónica, and Fernando 2021 [50]	28 Low SCD (14), High SCD (14)	Memory complaints questionnaire	Go/NoGo auditory-visual task	ERP N200 and P300 amplitude and latency	Lower amplitudes and longer reaction times in high SCD group	Small sample; No comparison with MCI, AD, controls
6. Tarantini, Bader, and Mecklinger 2021 [54]	29 SCD	MAC-S	Source memory test and visual oddball task	ERP P300 and LPP amplitude	Higher amplitudes with correct answers and deviant stimuli	Small sample
7. Stuckenschneider et al., 2020 [17]	26 SCD (13), MCI (13)	Self-reported complaints	Auditory oddball paradigm	ERP N200 and P300 amplitude and latency	No significant differences in task performance or ERP	Simple task, small sample
8. Cespón, Galdo-Álvarez, and Díaz 2018 [55]	34: low SCD (18), high SCD (16)	Memory complaints questionnaire	Simon task	ERP P300 and MFN amplitude and latency	P300 and MFN differences in high SCD during incompatible conditions	Small sample; Small difference in SCD levels
9. Laptinskaya et al., 2018 [51]	57 SCD (14), MCI (43)	Self-report question	Resting and silent video with auditory stimuli	Auditory ERP MMN and ΔMMN amplitude and latency	MMN: SCD ≈ MCI, ΔMMN associated with episodic memory	Small sample; No comparison with unimpaired or AD patients
10. Lazarou et al., 2018 [46]	57: SCD (14), MCI (17), AD (14), Controls (12)	SCD-I diagnostic criteria	Negative facial stimuli recognition	ERP N170 amplitude and latency; sLORETA	Reduced N170 amplitude in SCD group, similar to MCI and AD	Small sample; Negative emotions only
11. Idrizbegovic, Hederstierna, and Rosenhall 2016 [52]	103: SCD (27), MCI (44), AD (32)	Subjective complaints	Tone pulses task	Cortical auditory ERP MMN amplitude	MMN amplitude: MCI > SCD ≈ AD	Small sample
12. Hagen et al., 2015 [53]	358 SCD (54), Controls (304)	Self-report question	Vagus nerve stimulation	VSEP P100, P200, N200 amplitude and latency	Longer P200 latency in SCD	Small sample; Young and well-educated participants
13. Smart et al., 2014 [49]	40 SCD (17), Controls (23)	Self-report question	Go/NoGo attention task	ERP P300 amplitude and latency	Reduced P300 amplitude in SCD group	Small sample; Well-educated participants

Abbreviations: AD: Alzheimer’s Disease; ERP: Event-Related Potential; FRN: Feedback-Related Negativity; LPP: Late Positive Potential; MAC-S: Memory Assessment Complaints-Scale; MCI: Mild Cognitive Impairment; MFE-30: Memory Failures of Everyday-30; MMN: Mismatch Negativity; N170: N170 wave; P300: P300 wave; SCD: Subjective Cognitive Decline; sLORETA: standardised Low-Resolution Brain Electromagnetic Tomography; VSEP: Vagus Somatosensory Evoked Potential.

### 3.4. Fluid Biomarkers and Their Relation to EEG and ERP Biomarkers

The most widely used CSF biomarkers for AD are amyloid and tau proteins [56]. Pathological amyloid and tau profile have also been observed in MCI patients, with p-tau and t-tau proteins being the most accurate indicators of progression to AD [57]. A review by Lin et al. [58] presented conflicting results about CSF biomarkers in SCD participants. Several studies reported distinguishing Aβ42 and tau profiles in SCD participants relative to controls, MCI and AD patients, whereas others found no differences [58]. Other reviews [59] recognised the possible prognostic value of CSF biomarkers for indicating a clinical progression from SCD to MCI or AD.

**Figure 2:**
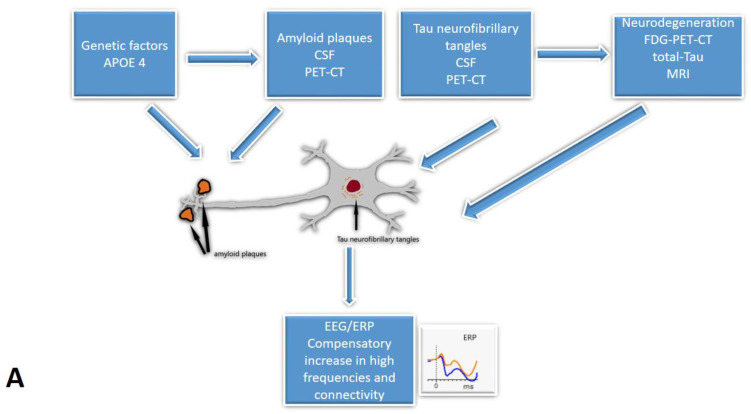

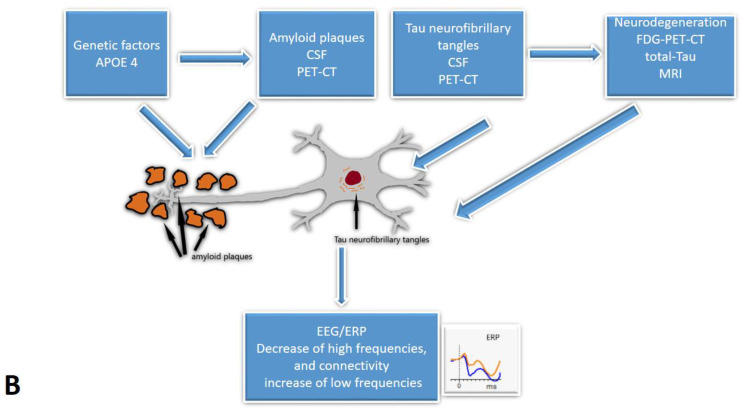
The cascades of developing Subjective Cognitive Decline: the relationship between EEG/ERP and other biomarkers: (**A**)—The early onset of Alzheimer’s disease, (**B**)—progression with amyloid beta accumulation. EEG/ERPs—Electroencephalography/event-related potentials; CSF—the cerebrospinal fluid.

Five studies [29,32,35,37,39] from our review included CSF analysis. The expected pattern of amyloid level decrease was shown when comparing SCD to MCI and MCI to the AD group. Tau proteins exhibited the opposite trend [29,32,35,37,39]. Furthermore, a few studies [35,37,39] indicated the relationship between CSF and EEG biomarkers. Smailovic et al. [35] described a negative association between CSF amyloid levels and GFP for delta and theta bands and an opposite correlation between CSF p-tau, t-tau, and GFP for alpha and beta bands. However, these trends were statistically significant only in MCI and AD groups. Moreover, Kramberger et al. [39] described slower background activity and a higher degree of episodic abnormalities associated with CSF biomarkers only in the AD/mixed dementia group. The authors tried to explain this by the heterogeneity of MCI and SCD samples [39]. Only Gouw et al. [37] reported higher relative theta and lower relative alpha and beta power in CSF amyloid-positive SCD subjects relative to the amyloid-negative SCD group. Gaubert et al. found two different EEG and connectivity patterns in regard to the amyloid load [31]. Alpha and beta frequencies and connectivity increase at the beginning of neurodegeneration until the amyloid load reach a certain threshold. At that point, delta and theta frequencies increase while alpha and beta frequencies and cortical connectivity decrease (Figure 2).

Plasma biomarkers are an emerging alternative to established CSF biomarkers. While CSF has the advantage of its close relation to the brain parenchyma, its use is limited by the invasiveness of sample collection. Blood samples are more easily accessible, and the procedure is less invasive than lumbar puncture. However, plasma biomarkers are affected by non-cerebral tissue secretions and the activity of proteases [56]. Besides plasma amyloid and tau proteins, other molecules, i.e., neurofilament light chain and micro-RNA, have shown diagnostic potential. Their levels seem to be altered in AD and MCI, and SCD participants [60]. More research is needed to investigate their diagnostic utility. None of the studies we reviewed provided data regarding plasma biomarkers. Their diagnostic value and relation to EEG/ERP biomarkers in SCD individuals would be interesting directions for future research.

### 3.5. Neuroimaging Biomarkers and Their Relation to EEG and ERP Biomarkers

#### 3.5.1. PET-CT Biomarkers

Besides fluid biomarkers, neuroimaging methods are a possible alternative for supporting the diagnosis of AD. A PET scan allows the assessment of amyloid and tau deposition and cerebral glucose metabolism. Progress of amyloid deposits in AD follows a specific pattern starting in the prefrontal and posterior parietal regions [61]. A similar distribution has also been observed in SCD individuals [62]. Higher amyloid burden at the baseline has been furthermore related to the faster cognitive decline of SCD [63].

Among 30 studies in our review, six [14,26,29,31,32,36] provided data about PET amyloid load. Shim et al. [26] noted increased delta and decreased alpha 1 activity in the amyloid-positive group of SCD participants. Similarly, Dubois et al. [32] described a decreased alpha-to-theta ratio and increased prefrontal alpha oscillations at the follow-up for SCD amyloid-positive participants. Two studies [31,36] investigated the relationship between EEG connectivity measures and amyloid load with conflicting results. Gaubert et al. [31] found a non-linear relationship between functional connectivity parameters and PET amyloid load, whereas Teipel et al. [36] found no association between phase-lag index and amyloid burden.

#### 3.5.2. MRI Biomarkers

Another category of neuroimaging biomarkers is MRI biomarkers. The most important structural parameters for assessing grey matter atrophy in AD are volumes of the hippocampus and entorhinal cortex [64]. For white matter, diffusion tensor imaging allows the assessment of microstructural changes reflecting degeneration [65]. Functional MRI provides information about brain activity and functional connectivity between brain regions, which are typically decreased in MCI and AD patients [66]. A large review study [63] on neuroimaging markers in SCD reported inconsistent and heterogenous results regarding structural and functional MRI parameters.

Two studies in our review [24,37] explored structural MRI biomarkers associated with ERPs. Ganapathi et al. [24] found a higher left temporal lobe volume in the SCD group compared to MCI and AD patients. Contrarily, medial temporal lobe atrophy did not differ between diagnostic groups in other research [37]. Ganapathi et al. [24] suggested a combination of MRI and ERP biomarkers. Diagnostic accuracy increased when temporal lobe volume was used together with P300 amplitude. Gouw et al. [37] reported better prognostic relative theta power value than the medial temporal lobe atrophy score. Although the data are limited, EEG/ERP biomarkers seem to hold the potential for both diagnostic and prognostic markers for pre-dementia stages and AD. They can be perceived as a promising, supplementary method to be used with other more established biomarkers.

EEG/ERP parameters have the advantage of being cheaper than neuroimaging biomarkers. They may also demonstrate brain activity in high time resolution (ms), while neuroimaging biomarkers provide excellent structural information [8].

### 3.6. Genetic Biomarkers and Their Relation to EEG and ERP Biomarkers

Despite the limited number of studies, some research has examined the association between ERPs and genetic biomarkers. Six studies [26,28,31,32,36,37] from our review included information on the apolipoprotein E (APOE) ε4 allele, a known risk factor for AD. All studies consistently reported a higher frequency of APOE4 genotype among the amyloid-positive SCD group relative to amyloid-negative SCD participants. Only in one study did authors try to establish a relationship between EEG parameters and APOE4 status. Gaubert et al. [31] found that APOE ε4 carriers had increased connectivity (wSMI) in comparison to non-carriers. This finding suggests that the presence of the APOE ε4 allele may be associated with alterations in ERPs and contribute to the risk of developing cognitive decline. Increased connectivity could be a compensatory mechanism for the neurodegeneration at the very beginning of AD.

### 3.7. Pharmacological Intervention

Five studies [23,29,30,33,53] reported types of medication when describing the characteristics of their sample, although some studies excluded patients who received neuroleptic drugs or any other drug that could cause drowsiness [52].

The frequency of drug use was generally higher among AD and MCI participants than in the SCD group. The most common medication were benzodiazepines, antidepressants, and hypnotics. Only in one study did patients receive anti-dementia drugs [30].

Since SCD lacks an established, standardised therapeutic intervention, the treatment modalities varied across different studies. In their systematic review, Bhome et al. [67] reported that some studies explored dietary supplements’ effects, while most focused on cognitive training. Despite these interventions, none significantly enhanced overall cognitive performance [67]. A notable observation from our review was a study that used tricyclic antidepressant treatment, despite the potential risk these drugs carry of exacerbating memory conditions [53].

Within our review, only one study [29] explored the potential influence of acetylcholinesterase inhibitors, benzodiazepines, anti-epileptic drugs, and antidepressants on cortical connectivity and EEG patterns. A modest but significant effect was observed with the use of antidepressants on EEG, characterised by a decrease in the beta frequency band and an increase in the delta frequency band. Therefore, we need more studies to better understand the therapy’s effect on EEG/ERPs.

### 3.8. Limitations

Our review, encompassing 30 studies, offers insights into EEG and ERP biomarkers in individuals with SCD. While some findings are consistent across the studies, others present conflicting results. Several factors may contribute to these inconsistencies. In some cases, the number of SCD subjects was relatively small [17,34,51]. Although human neurophysiology studies typically involve small sample sizes (between 10 and 25 participants), these disparities may lead to discrepancies in the findings [68]. Vozzi et al. [69] proposed a threshold of 24 participants, below which the results begin to lose statistical power. The 30 studies in our review employed varying diagnostic measures for SCD, which influenced the inclusion criteria for participants. The EEG measuring conditions were relatively controlled (resting-state condition or established cognitive task). However, different EEG systems, electrode numbers, and analysis software were utilised. These factors may result in conflicting outcomes and limit their generalizability.

Most studies used SCD subjects as a control group compared to patients with either MCI, AD, or other types of dementia. Only ten studies contrasted EEG and ERP biomarkers in SCD participants with control subjects without SCD; only 4 out of 30 analysed studies were longitudinal.

## 4. Conclusions

Our review explores the use of EEG and ERP biomarkers in patients with SCD. As unique tools, EEG and ERPs offer real-time insights into cortical activity, setting them apart from other biomarkers. Some studies have demonstrated the association between EEG/ERPs and neuroimaging, CSF, and genetic biomarkers, while others have not.

Combining results from the studies included in our review, it may be possible that during the early onset of Alzheimer’s disease, cortical activity frequency increases as part of a compensatory mechanism, enhancing connectivity. However, once the amyloid accumulation reaches a certain point, beta frequencies tend to decline, and the power of slower theta and delta frequencies rises.

In the future, more in-depth, longitudinal studies are needed. These should incorporate neuroimaging and CSF biomarkers, extend to plasma biomarkers, and potentially explore therapeutic impacts on cortical activity.

## Figures and Tables

**Table 1:** Keywords used and number of results in PubMed and Web of Science databases.

Keywords		Nr. of Results in PubMed	Nr. of Results in Web of Science
subjective cognitive decline AND	electroencephalography OR	476	70
	event-related potentials OR	270	42
	event-related synchronisation desynchronisation	8	0
subjective cognitive impairment AND	electroencephalography OR	587	118
	event-related potentials OR	337	68
	event-related synchronisation desynchronisation	7	0
subjective memory complaint AND	electroencephalography OR	26	39
	event-related potentials OR	17	21
	event-related synchronisation desynchronisation	0	0

## Data Availability

The data presented in this study are available on request from the corresponding author.
